# Psychological Health and Physical Activity Levels during the COVID-19 Pandemic: A Systematic Review

**DOI:** 10.3390/ijerph17249419

**Published:** 2020-12-15

**Authors:** Verónica Violant-Holz, M. Gloria Gallego-Jiménez, Carina S. González-González, Sarah Muñoz-Violant, Manuel José Rodríguez, Oriol Sansano-Nadal, Myriam Guerra-Balic

**Affiliations:** 1 Faculty of Education, Universitat de Barcelona, 08035 Barcelona, Spain; 2 Faculty of Education, Universidad Internacional de la Rioja, 08014 Barcelona, Spain; gloria.gallego@unir.net; 3 Department of Computer Engineering and Systems, Universidad de la Laguna, 38293 La Laguna, Spain; cjgonza@ull.edu.es; 4 Department of Psychology, The University of British Columbia, Vancouver, BC V6T 1Z4, Canada; munozvio@mail.ubc.ca; 5 Department of Biomedical Sciences, School of Medicine and Health Sciences, Institute of Neurosciences, Universitat de Barcelona, 08036 Barcelona, Spain; marodriguez@ub.edu; 6 Faculty of Psychology, Education and Sports Sciences, University Ramon Llull, Spain FPCEE-Blanquerna, 08022 Barcelona, Spain; oriolsn@blanquerna.url.edu (O.S.-N.); miriamelisagb@blanquerna.url.edu (M.G.-B.)

**Keywords:** COVID-19, physical activity, mental health, psychological distress, coronavirus, lockdown, pandemic

## Abstract

The coronavirus disease (COVID-19) pandemic has been devastating in all senses, particularly psychologically. Physical activity (PA) is known to aid psychological well-being, and it is worth investigating whether PA has been a coping strategy during this pandemic. The objective of this literature review is to analyze the extent to which engaging in PA during the COVID-19 pandemic impacts psychological health in the adult population. The literature was searched in all databases from the EBSCOhost Research Database—MEDLINE, APA PsycArticles, between others—published between 1 January 2019 and 15 July 2020. From 180 articles found, 15 were eligible. The reviewed articles showed an association between mental health distress—e.g., stress, anxiety, depressive symptoms, social isolation, psychological distress—and PA. This research concludes that the COVID-19 pandemic and the lockdown measures caused psychological distress. Those studies that analyzed PA showed that, during quarantine, adults increased their sedentary time and reduced their PA levels, showing controversial psychological outcomes. This review discusses whether PA is an effective strategy to face the COVID-19 pandemic psychological effects contributing to a further putative increase in the prevalence of psychiatric disorders.

## 1. Introduction

The coronavirus disease (COVID-19) is a pathology induced by a new coronavirus, severe acute respiratory syndrome coronavirus-2 (SARS-CoV-2) [1]. To date, there is no effective treatment for this infection [2]. Therefore, prevention measures such as frequent hand washing, keeping physical distance, and wearing face masks have been widely implemented. Following World Health Organization (WHO) recommendations [3], most countries are implementing lockdown policies for all citizens except for essential services.

This pandemic has not affected everyone equally. A WHO compilation of multiple studies [4] reports a higher risk of acute symptoms and death by COVID-19 in people with underlying conditions such as hypertension, cardiopathies, pneumonia, and cancer. Age is another important factor, with the elderly population being at higher risk for the disease, while children are less likely to develop acute clinical states [4].

Researchers have conducted multiple investigations of the consequences of the disruptive routine changes experienced by most individuals due to the COVID-19 pandemic. Some common impacts include disturbed eating behaviors such as increased comfort food consumption [5], eating in response to stress and boredom, a snacking after dinner [6], decreased physical activity (PA) [7], and either significant increases [8] or reductions [7] in alcohol consumption.

An important consequence of this pandemic has been the global psychological distress; multiple researchers have found increased prevalence of pandemic-related psychiatric morbidity and psychological distress [9,10]. The higher prevalence of anxiety and stress-related disorders may be purely pandemic related, such as fears that oneself or a loved one will contract the virus, and generalized uncertainty about the future [11], but the direct biological effects of the virus itself on the central nervous system (CNS) are unknown. A meta-analysis from previous coronavirus infections revealed that common symptoms during the acute phase of the infection were depressed mood, anxiety, confusion, and impaired memory. If SARS-CoV-2 follows a similar course to that of previous coronaviruses, patients should recover from psychiatric symptoms without experiencing mental illness [12].

The act of quarantining itself adds a facet to mental health deterioration [9]. For example, anxiety and depression prevalence almost doubled in participants who had to quarantine or whose friends and family had to quarantine compared with participants who did not [13]. Consequently, the first question that we aimed to answer with this study was the following: (1) What psychological impact has the COVID-19 pandemic had on adult populations?

In one systematic review, cultivating healthy coping strategies and resilience during the pandemic were major challenges within the study population [14], suggesting that exploring strategies to improve psychological well-being during lockdown is necessary, even as it is challenging as well. One possible coping strategy is PA: In a previous study with more than 1.2 million participants, some individuals who exercised presented fewer days of poor mental health in the previous month than did participants who did not exercise, and this association between exercise and mental health burden was found across age, gender, race, household income, and types of exercise [15].

Researchers have also investigated how the COVID-19 pandemic has affected PA and the consequences for mental health. Lockdowns have limited individuals’ to engage in outdoor exercise, forcing people to exercise at home, which could explain why some studies have reported a negative effect on exercise engagement [7,16], although overall findings have been mixed. For example, in a study with university students, PA had mostly decreased, but a significant portion of the sample was able to maintain and even increase their exercise practice [17]. Furthermore, in another study, PA decreased during the first week of confinement and gradually increased as individuals adjusted to the lockdown [18]. This conflict led to the second question of this review: (2) What impact has the COVID-19 pandemic had on PA levels in adult populations?

Questions (1) and (2) led us to explore the relationship between PA and mental health; therefore, our final question was the following: (3) Does PA affect psychological health during the COVID-19 pandemic in adult populations?

To answer these research questions, we conducted a systematic review with the objective of analyzing the extent to which engaging in PA during the COVID-19 pandemic affected adults’ psychological health.

## 2. Materials and Methods

### 2.1. Design

This article focuses on peer-reviewed journal articles about the impacts of PA on mental health during the COVID-19 pandemic that were published between 1 January 2019, and 15 July 2020.

### 2.2. Databases and Search Strategy

The EBSCOhost Research Database was searched on 15 July 2020, to locate studies in APA PsycArticles; eBook Subscription Harvard Business Publishing Collection Trial; Art & Architecture Source; CINAHL Complete; eBook Collection (EBSCOhost); E-Journals; ERIC; Film & Television Literature Index; GreenFILE; Library Literature & Information Science Index (H.W. Wilson); Library Literature & Information Science Retrospective: 1905–1983 (H.W. Wilson); Library, Information Science & Technology Abstracts; MEDLINE; Teacher Reference Center; MLA Directory of Periodicals; MLA International Bibliography with Full Text; APA PsycInfo; Psychology and Behavioral Sciences Collection; PSICODOC; Consumer Health Main Edition; Sales & Marketing Source].

**Figure 1:**
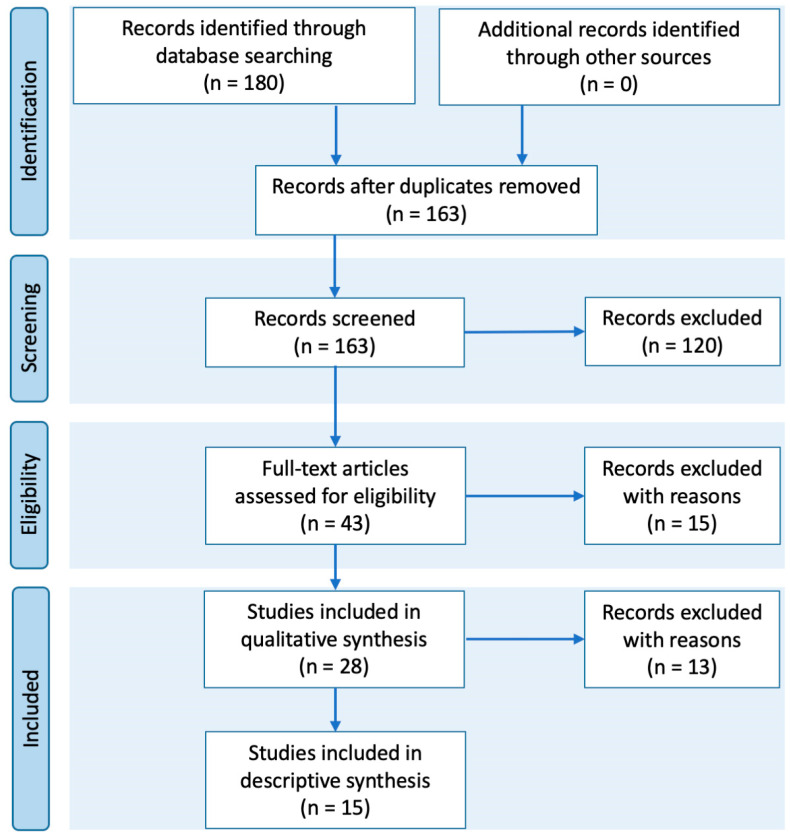
Flow diagram of study selection process.

An advanced search was conducted using these terms: COVID-19 AND ((“psychology” or “psychological” or “mental health” or “depression” or “anxiety”)) AND ((“physical activity” or “exercise” or “fitness” or “physical exercise” or “sport”)). The flow diagram was created according to the Preferred Reporting Items for Systematic Reviews and Meta-Analyses (PRISMA) [19] (see Figure 1).

### 2.3. Inclusion/Exclusion Criteria Selection of Studies

To ensure the reliability of the systematic review, three authors independently completed an inclusion/exclusion checklist while screening the titles, keywords, and abstracts of the primary search. A qualitative analysis was conducted using consensus agreement to resolve disagreement during three separate sessions [20,21].

The articles reviewed were screened by title, keyword, and abstract and then classified into three categories: (a) articles excluded for meeting exclusion criteria; (b) articles excluded for only partially meeting inclusion criteria or for only being associated with mental health or PA rather than both; or (c) articles included because all inclusion criteria were met (Table 1). The full texts of the latter group were analyzed with the aim of establishing whether PA has affected psychological health among adult populations during the COVID-19 pandemic.

## 3. Results

This search resulted in 180 articles in seven databases (Table 2). After cleaning for duplicates, 163 articles remained. Based on the criteria, we excluded 120 articles (in red). From the remaining 43 articles (in yellow or green), 15 only referred to one of the two key words (i.e., “mental health” or “PA”). Excluding these left 28 articles for full-text analysis (see Figure 1).

The excluded articles are presented in detail below:

(a) From the 120 initially excluded articles: 1. The study could not be retrieved (0); 2. The study is within the theoretical frameworks of medicine (1); 3. The study population is: 3.1. pregnant women, people with physical disabilities, and/or prisoners (2); 3.2. individuals who are not yet adults (12); 3.3. participants who suffer from a particular illness or patients with COVID-19 or history of the disease (38); 4. The type of publication was report (14), opinion study, political commentary, social media events, and protocols (15), essays (11), case studies (6), reviews, systematic reviews, or meta-analysis (5), no original research (16);
(b) From the second round, 15 articles were excluded because they were associated with mental health (7) or PA (8) only but not both;
(c) From the third round, 13 articles were excluded after reading the full text because they were reports (7); report reviews (3); commentaries (1); or protocol research (2).

All the papers reviewed included adult participants: eight from general populations, three with college or university students, one with young adults, one with elite athletes, and two of health care professionals, a total of 15. Most studies comprised both female and male participants and had higher proportions of women; the exceptions were [22], a study of women only, and [23], in which the authors did not mention a female/male ratio.

Regarding the research design, all articles used a cross-sectional methodology except [22,24,25,26], which were longitudinal prospective studies. Studies came from Europe, America, Asia, Africa, and Australia, and participants were recruited primarily through snowball sampling in online social media and through newspapers; three studies did not state the recruitment method [22,27,28]. It is worth mentioning that [25] developed and evaluated an intervention.

The instruments applied in the reviewed papers were questionnaires, most of which had already been validated and previously translated into the respective native languages. There are studies that used ad hoc survey, such as [29], and some others such as [30] used questions. The data source in one study was data from video conference interviews with focus groups [25]. Authors of the studies used ad hoc questionnaires to obtain sociodemographic information such as age, gender, marital status, location, ethnicity, level of education, kind of job, household, number of people living together, smoking, and chronic diseases.

Investigators used a wide range of questionnaires, most commonly the IPAQ [26,31,32], PSQI [26,27], and DASS-21 [26,33]. Authors also used the SF-12 [28], SAS [27], SDS [27], and PCL-C [27]. In some studies, authors tied COVID-19 symptoms to specific data such as number of patients or number of deaths and how these had affected the population; the authors had obtained these particular data from official documents [22,26]. One paper was a description of an eight-week intervention during the COVID-19 lockdown. The authors used video-based modules to reduce depression, stress, and anxiety [33].

The most commonly reported mental health symptoms were anxiety, depression, and sleep problems. Women tended to be more vulnerable and reported higher stress levels [30,31,33]. Moreover, young participants seemed to experience enhanced long-term well-being even though they faced more changes in their daily routines and these had caused higher levels of anxiety, distress, and depressive symptoms [33]. Concerning PA, studies showed decreases in participants’ number of hours and in intensity of exercise. Overall, investigators measured severity of the lockdown based on diet, loneliness, online work, environment, financial status, and outdoor options for exercise.

The first question that this review aimed to answer, regarding psychological impacts of COVID-19 on adult populations, was only addressed by one study that focused on mental health issues; this was the study on applying an intervention to reduce symptoms of depression, anxiety, and stress [25]. However, multiple researchers discuss other direct or indirect influences on mental health such as feelings of loneliness and isolation. For example, in one study, participants who lived alone during lockdown showed higher levels of psychoticism [23], and in another, even individuals who lived in households of two to four people reported total or partial isolation [29]. Notably, feelings of isolation and the subsequent impacts on stress levels were highly related to education levels, gender, income brackets, and housing conditions. Moreover, feelings of loneliness were particularly high among nurses, advanced practice providers, and home care staff [30].

Sleep during the COVID-19 pandemic was another direct influence on mental health: In one study, 56% of the sample reported some change in sleeping patterns, with around 26% sleeping more and 31% sleeping fewer hours than before the pandemic [29]. Furthermore, authors of two other studies also found poor sleep quality in their samples [6,26]. Again, health care providers, in particular nurses, physicians, and advanced practice providers, domestic staff reported the worst changes in their sleep-wake cycles and routines [27,30,33,34].

The second question was regarding levels of PA among adult populations during the COVID-19 pandemic. Authors of [6] focused on issues such as weight gain during the pandemic and found that only 59% of participants had remained weight stable (22% had gained and 19% had lost weight); snacking and general lack of dietary restraint, eating when stressed, and low PA were risk factors for weight gain during lockdown. Focusing on athletes, [34] found that diets became poorer during the lockdown, with greater carbohydrate intake. Additionally, PA mediated the relationship between COVID-19 pandemic severity and life satisfaction [28].

The third research question, (3) does PA affect psychological health during the COVID-19 pandemic in adult populations?, was addressed in several studies in which authors tied mental health to PA [23,24,27,32]. Some authors did not analyze the relationship between the two concepts but rather studied PA and mental health separately [30,33], but some authors extended their analyses to consider other variables and better understand behavior during lockdown [35]. Variables included the working status of participants [33] correlated to age, sex, education level, income level, and toxic habits (smoking and alcohol consumption) [29], and socioeconomic status and educational background were particularly influential. For instance, in [33], respondents showed lower depression scores as years of education increased, but the same did not apply for anxiety and distress levels. Furthermore, individuals with higher education levels and incomes reported that social interaction had been the most affected aspect of their lives, while participants with low incomes and less education reported greater financial impacts [29]. Indeed, in another study, 32% of the sample had been laid off due to the pandemic and 20% had had their working hours reduced [6].

In another study [22], the authors did not study PA directly but found that after two weeks of lockdown, sedentary behavior of participants increased. Finally, some authors only obtained information about PA as a coping strategy but did not get information regarding PA type or level [30], evaluated the importance of exercising and sleeping adequately [26], or focused on physically active people whose jobs had been affected by the pandemic and who had consequently stopped engaging in PA as frequently as they previously had [28].

Other coping strategies study participants reported using to support resilience included yoga, meditation, virtual support groups, and talk therapy [30]. In particular, religion and spirituality had helped participants with a sense of meaning during the COVID-19 pandemic. At the same time, participants reported maladaptive coping behaviors such as drinking alcohol and smoking tobacco [33]. The main characteristics of the eligible studies are summarized in Table 3, and the definitions, measurement, and outcomes are presented in Table 4.

## 4. Discussion

This review focused on the impacts of the COVID-19 lockdown specifically on mental health, although we also provide some evidence that PA can be considered as a strategy to improve daily life during the quarantine. In nearly all of the studies that comprised our systematic literature review sample, investigators used online surveys as the main instrument. One important finding we identified was serious employment and financial changes resulting from the pandemic [6,29]. For instance, we found that individuals who were actively working showed fewer depression symptoms than did unemployed people [23], unless they were medical staff [36] who treated patients with COVID-19 [37]. This has important consequences for vulnerable groups such as younger adults and women [23,31,33].

Regarding mental health, the most analyzed variables were aggressiveness and hostility [23,24,26], depression [22,32,34], anxiety [23,33,35], stress [24,25,29], sleep [26,29,31], and nutrition [6,28,31,34]. Other analyzed variables were lifestyle habits and satisfaction [22,28,31], alcohol intake and cigarette smoking [33], social isolation [29], and distress and coping behaviors [30]. In all cases and across all populations, pandemic-related changes were steadily negative until they improved with PA.

Anxiety was more frequent in individuals with relatives and close others diagnosed with COVID-19 than it was in persons who did not know anyone with the virus [23]. Women, young adults, and people with chronic illnesses also showed higher levels of anxiety, stress, and depression [33]. In several studies, the pandemic had increased perceived stress across cohorts [24,25,29], and the quality of sleep decreased as well [26,29,31]; in particular, knowing the number of COVID-19 deaths showed a negative impact on sleep quality [29,33]. Researchers argue that these changes are related to the social isolation and drastic changes in lifestyle and financial and occupational health caused by the pandemic.

Although the lockdown measures implemented worldwide reduced the spread of COVID-19 [29], they had undeniable negative impacts. People who lived alone showed higher levels of psychoticism [23,29], and loneliness in older adults accelerated physical and cognitive decline [38] while social isolation increased hostility and anger levels in young adults [23,24].

Findings show several benefits of PA [39,40]; for example, people who exercised daily presented fewer somatization symptoms, lower stress levels, and more normal sleep than did individuals who did not exercise [29,35]. Moreover, PA of adequate intensity and quantity releases psychological tension and increases mental stability [27], and researchers found detrimental effects associated with lack of PA, specifically greater anxiety, depression, and stress [22,31,33,41]. Unfortunately, the pandemic has negatively affected in PA, in particular in outdoor activities, which have been shown to have protective effects for well-being [35].

Furthermore, COVID-19 was associated with increased phone usage, decreased PA, locations visited, and increased time spent watching pandemic-related news [22,24,30]. In one study, the authors used a smartphone app with sensors to capture locations visited, sedentary behavior time, travel lengths, phone’s usage, number of phone unlocks, and sleep length [22]. However, there is some controversy around the use of these kinds of measures related to privacy and data protection laws. Notably, being disconnected from workplace or education demands and spending more time with family and friends or on hobbies contributed to well-being [24]. In the only study that applied an intervention, depression, stress, and anxiety levels decreased after eight weeks of a web-based mindfulness virtual community program and cognitive behavioral therapy [25].

Regarding the research questions for this study, we address them below.

(1) What psychological impact has the COVID-19 pandemic had on adult population?

The COVID-19 pandemic and the lockdown caused stress, anxiety, and mental distress in adult populations around the world. Psychologically vulnerability was conditioned by sociodemographic and employment context factors [23]. For example, young adults experienced increased levels of perceived stress and anger [24].

In a study of medical COVID-19 staff, front-line workers showed greater somatization, depression, and anxiety than usual in addition to reporting poor sleep quality and feelings of fear and terror associated with performing tasks that required exposure to unknown conditions [27]. Other authors focused on social isolation, which is a risk during lockdown periods, and found that social isolation tended to reduce PA and correlated with unhealthy diet, depression, anxiety, and stress [41]. Moreover, in one study, a third of the respondents reported social interaction as the most affected factor during the lockdown [29]. Finally, lockdown has worsened neuropsychiatric symptoms among individuals with preexisting central nervous system diseases. For example, individuals with Alzheimer’s disease and mild cognitive impairments showed increased apathy, unusual motor activity, and agitation [42].

(2) What impact has the COVID-19 pandemic had on PA levels in the adult populations?

PA produces many benefits related to improved mental health as well as bone and muscle health, better weight management, and decreases in certain diseases [35,36]. At the same time, the risks for non-communicable diseases and all-cause mortality increase when PA levels are low [43]. It is important to encourage individuals to increase their PA and decrease their sedentary behaviors because evidence shows that being sedentary can be harmful to health even if one is otherwise successfully meeting the recommended moderate-to-vigorous PA guidelines [44,45].

Other researchers found observable effects of quarantine on cardiovascular morbidity, mainly due to poor diet habits as well as decreased PA [41,46]. In one study, only 40% of adults were doing some kind of exercise during lockdown [29], and in another, college students were more sedentary, anxious, and depressed during the 2020 winter term, and sedentary behavior increased during the second week of the break [22]. Some studies showed negative impacts of the lockdown on mental health and physical activity [47,48,49]. Some researchers investigated pandemic-related stages of change in exercise behavior based on the transtheoretical model of behavior change [50], and participants who were at a negative stage of change during the early COVID-19 restrictions showed poorer mental health and well-being than did participants in other change stages [51].

(3) Does PA affect psychological health during the COVID-19 pandemic in adult populations?

Authors of one review analyzed several studies related to lockdown periods following upper respiratory tract viruses [52] and found several factors associated with health risks such as smoking, diet, and psychological stress. In contrast with these, PA, social integration and support, good sleep quality, and mild alcohol intake seemed to reduce health risks. However, these results should be considered cautiously and might not generalize to the COVID-19 pandemic because the lockdown periods under study only lasted five to six days.

Study participants had received recommendations for taking care of their psychological and physiological as well as social health to avoid frustration and other negative emotions, and one of these recommendations was to control their nutrition and be physically active. Different entities offered a wide variety of online resources to achieve these goals. In an interesting longitudinal study, strategies for potentially improving mental health included daily PA and proper sleep hygiene [26]. Older adults experienced particularly negative impacts because PA programs for the elderly were severely curtailed for fear of rapid infection spread among this vulnerable population. Confinement produces negative consequences such as increased sedentary behavior, which is particularly detrimental for the elderly; one study’s authors also found that proposed alternatives for exercising at home were sometimes less effective and that social networks were essential in encouraging older adults to exercise [38]. Moreover, feelings of loneliness could accelerate physical and cognitive decline, a conclusion supported by a finding that exercise interventions have minor clinical impact on PA in older adults [53].

In one PA intervention, the observed improvement had faded six months after the intervention ended [54], and authors of another study identified no clinical benefits from PA interventions for older adults [26]. It might be noteworthy that benefits could have been affected by lacks of necessary equipment for exercising at home along with insufficient frequency of PA. For example, in [29], stress levels and sleep quality improved in people who exercised regularly, and another study showed a decreased risk for depression and anxiety symptoms in participants who reported ≥30 min of moderate-vigorous PA/day; in contrast, participants in that day who spent ≥ 10 h a day sedentary were more likely to present depressive symptoms [55].

Finally, in [35], the authors found significant differences among participants whose activity levels after COVID-19 had increased from before, stayed the same, or decreased, although levels of generalized anxiety between active and inactive participants were not significant. The authors concluded that public health restrictions influenced PA, particularly outdoor PA, which seems to offer protective factors for well-being. The authors of [30] found that exercising was the most commonly adopted coping strategy during the COVID-19 lockdown.

Authors of nearly all of the studies in this review used online surveys as their main instrument, and this presents an important limitation: Individuals with limited access to technological devices face severe limitations in the ability to participate in such surveys, for instance persons of low socioeconomic status and older persons unaccustomed to new technologies. Furthermore, the sample populations of some studies showed little diversity, and results should be interpreted with caution in that respect as well. For example, most participants in one study were undergraduate students, and 82% considered their homes good or excellent, and housing conditions have been a key factor in the psychological impacts of the lockdown. For instance, persons with spacious homes and grounds have more freedom to exercise outdoors and might thus have experienced less severe outcomes from the lockdown than those experienced by individuals who had to spend 20–24 h/day inside such as in [6]. Finally, it is important to consider that new studies are constantly ongoing because so many COVID-19 impacts remain unknown and the pandemic continues to be a matter of concern for both the public and the scientific community.

The existing literature highlights a need to develop strategies for coping with stress in isolation. Internet-based mindfulness CBT-based interventions have been shown to reduce symptoms of depression and anxiety [25]. These tools are widely available and accessible to general populations and could reduce sedentary behavior and aid coping. Future researchers should focus on PA as a coping strategy against the negative consequence of psychological distress caused by the COVID-19 pandemic given that PA has been shown to be an effective strategy against mental health decline [56].

## 5. Conclusions

This review summarizes the existing evidence on the impacts of the COVID-19 pandemic on psychological well-being among adult populations and the effects of physical activity on psychological health during the same period of time. The findings we reviewed in this study reflect that the pandemic and the lockdown measures caused stress, anxiety, social isolation, and psychological distress in adults and higher than usual depression and anxiety levels in front-line medical staff. In some analyses, adults grew more sedentary during quarantine and decreased their PA levels, with sometimes detrimental psychological outcomes. Further studies are necessary to clarify whether PA is an effective strategy for coping with negative psychological effects of the COVID-19 pandemic. Indeed, a detailed analysis of these strategies would help to establish the utility of PA for preserving psychological well-being during this exceptional and psychologically harmful period of our lives. This review could be of interest to institutional leaders and governments, as well as to health professionals and researchers, for informing clinical decisions and policies for future pandemics.

## Figures and Tables

**Table 1:** Inclusion and Exclusion Criteria.

Inclusion Criteria	Exclusion Criteria
1. Empirical research and peer-reviewed articles	1. The study could not be retrieved.
	2. The study is within the theoretical frameworks of biomedicine, genetics, and pharmacy.
2. Study population: 2.1. Participants are aged 18 or older	3. Study population: 3.1 Participants are pregnant women, people with physical disabilities, and/or prisoners 3.2. Participants are individuals who are not yet adults (infants, children, teenagers) 3.3. Participants suffer from a particular illness
3. Time period: 3.1. Published from 1 January 2019 to 15 July 2020	4. Type of publication contains no original data such as reports, opinion studies, essays or comments, case studies, reviews, and no research.
4. Publication criteria: 4.1. Written in English and/or Spanish 4.2. Any country 4.3. Have an abstract	
5. Type of publication contains: lifestyle habits, anxiety levels, association between psychological distress and changes in health behaviors, preservation of PA during the pandemic, obstacles and facilitators of PA, psychopathological symptoms, impact of the COVID-19 outbreak	

**Table 2:** Literature search results.

Databases	Articles
MEDLINE	107
APA PsycInfo	60
CINAHL Complete	6
Psychology and Behavioral Sciences Collection	3
Library, Information Science & Technology Abstracts	2
APA PsycArticles	1
GreenFILE	1

**Table 3:** Main Characteristics studies.

Authors	Setting of Target Population	Study Design	Sample Size	Age Range	Gender/Sex	Population	Recruitment
Ahmad, 2020 [25]	Canada	Intervention	*n* = 113	---	M = 28 F = 85	Undergraduate	Online
Antunes, 2020 [31]	Portugal	Cross-sectional design	*n* = 1404	18 and older	F = 977 M = 426 Prefer not to specify = 1	General population	Social media and newspapers
Becerra-García, 2020 [23]	Spain	A cross-sectional pilot study	*n* = 151	18–76 years	---	General population	Online
Huckins, 2020 [22]	United States	Longitudinalmultimodal study	*n* = 217	18 to 22 years	F = 147 M = 70	University students	Not mentioned
Lesser, 2020 [35]	Canada	Cross-sectional design	*n* = 1098	19 and older	F = 871 M = 215	General population	Twitter, Facebook, and LinkedIn; local newspaper; national news network
Maugeri, 2020 [32]	Italy	Cross-sectional design	*n* = 2524	18 and older	F = 1426 M = 1098	General population	Instagram, LinkedIn, Facebook, WhatsApp, and email
Pillay, 2020 [34]	South Africa	Cross-sectional study	*n* = 692	18 and older	F = 225 M = 463 Prefer not to say = 4	Semi-elite athletes	WhatsApp
Shanahan, 2020 [24]	Zurich	Prospective-longitudinal study Type: Longitudinal cohort study	*n* = 1180 *n* = 786	20–22 years	---	Young adults	Online survey
Shechter, 2020 [30]	United States	Cross-sectional design	*n* = 657	18 or older	F = 509 M = 143 Genderqueer = 1 Prefer not to answer r = 4	Healthcare workers	Email
Stanton, 2020 [33]	Australia	Descriptive statistics; Non-parametric analysis: Kruskal–Wallis test, Wilcoxon rank-sum, and Spearman’s correlation; Linear regression; Logistic regression; Crude odds ratios	*n* = 1491 Sample: 1491 adults	18–45 years	F = 484 M = 999	General population	Online survey (Qualtrics survey platform) Social media sources and institutional outreach
Vasconcelos, 2020 [29]	Brazil	Cross-sectional design	*n* = 16.440	18 or older	F = 11344 M = 5096	General population	WhatsApp, Instagram, and Facebook
Wu, 2020 [27]	China	Experimental design	*n* = 120	25–59 years	F = 89 M = 31	Front-line clinicalstaff	Not mentioned
Zachary, (2020) [6]	United States	Quantitative descriptive/correlational design	*n* = 173	18 or older	F = 96 M = 77	General population	Facebook
Zhang, 2020a [26]	Multiple provinces across China	Longitudinal survey design	*n* = 66	Average = 20.70 years	F = 41 M = 25	University students	WeChat moments and WeChat pushes
Zhang, 2020b [28]	China	Cross-sectional design	*n* = 369	Average = 36.6 years	F = 165 M = 204	General population	Not mentioned

**Table 4:** Goals, measurements, and outcomes.

Authors.	Goals	Measurement (Instruments)	Outcomes
Ahmad, 2020 [25]	To study the MVC ^a^ intervention and its effects in depression, anxiety, stress, quality of life, life satisfaction, and mindfulness.	Patient Health Questionnaire-9 item Beck Anxiety Inventory Perceived Stress Scale Quality of Life Scale Brief Multi-Dimensional Students Life Satisfaction Scale Five-Facet Mindfulness Questionnaire	Mental Health ○ Online CBT ^b^–based mindfulness interventions significantly reduce symptoms of depression, anxiety, and stress.
Antunes, 2020 [31]	To observe lifestyle habits, anxiety levels and BPN ^c^ during the COVID-19 ^d^ pandemic.	IPAQ ^e^ Basic Need General Satisfaction Scale State-Trait Anxiety Inventory	Mental Health: ○ Quarantine led to higher levels of stress, anxiety, and mental distress. ○ Women showed higher anxiety states and anxiety traits. ○ The 18–34 age group showed higher anxiety scores for total energy expenditure and satisfaction of competence. PA ^f^: ○ Men showed higher values for total energy expenditure and for satisfaction of competence. ○ Confinement may cause people to practice less PA.
Becerra-Garcia, 2020 [23]	To analyze psychopathological symptoms during the COVID-19 quarantine based on sociodemographic, occupational, and environmental-contextual variables.	The Symptom Assessment-45 Questionnaire Ad hoc questionnaire	Mental Health: ○ Younger participants showed higher levels of hostility, depression, anxiety, and interpersonal sensitivity than did older participants. ○ People show were actively working showed lower depression scores than did unemployed individuals. ○ Those who were less informed about COVID-19 showed higher scores for hostility and interpersonal sensitivity. ○ Those who knew people affected by COVID-19 presented higher levels of anxiety. Mental health association with PA: ○ Participants who performed daily PA showed less somatization.
Huckins, 2020 [22]	To study behaviors and mental health during the COVID-19 pandemic. To study the relationship between COVID-19 news and mental health changes.	Patient Health Questionnaire-4: includes depression and anxiety Assessed using weekly self-reported EMAs ^g^. GAD-2 ^h^ Student Life smartphone sensing app	Mental Health: ○ During lockdown, individuals were more sedentary and presented more anxiety and depression symptoms. ○ Anxiety was significantly associated with the COVID-19 news. PA and sedentary behavior: ○ Time spent sedentary increased through the second week of the break. ○ When analyzing the effects for phone usage, all variables except sleep time and distance traveled were significantly associated with the number of COVID-19 news.
Lesser, 2020 [35]	To study the impacts of the COVID-19 pandemic and public health constraints. To report changes in PA obstacles, facilitators, engagement, and well-being. To investigate differences in outdoor PA.	GAD-7 ^i^ Mental Health Continuum Godin Leisure Questionnaire BREQ-3 ^j^ Outdoor PA NRS ^k^	PA: ○ Both active and inactive individuals became less active. ○ Individuals participated in PA at home or in their neighborhoods. ○ Active participants spent significantly more time in outdoor PA. ○ Measures like benefits, enjoyment, confidence, support, and opportunities were smaller for inactive participants. ○ PA engagement was a challenge for inactive participants. ○ Active participants had more motivation and external regulation than did inactive individuals. Mental health related to PA: ○ The level of anxiety affects PA amount and outdoor PA. ○ Participants who engaged in PA with others had higher mental health scores.
Maugeri, 2020 [32]	To study changes in PA levels during the lockdown. To analyze the impacts of PA on mental health.	IPAQ-SF ^l^. Psychological General. PGWBI ^m^.	PA: ○ Participants with low PA before the pandemic increased their activity during the COVID-19 lockdown. ○ Highly and moderately active participants exercised less during the lockdown. ○ Total PA significantly decreased across age ranges. Mental health related to PA: ○ Women and participants with low activity showed moderate distress. ○ Low PA was associated with worse mental well-being.
Pillay, 2020 [34]	To study athletes’ perceptions on returning to their sports. To investigate maintaining PA during the pandemic. To study the athletes’ knowledge of COVID-19, mental health, sleep, health care access, and nutrition.	PAR-Q ^n^. REAP-S ^ñ^. Personality trait model and inventory for DSM-5 ^o^.	Mental Health: ○ Depression appeared in many athletes. ○ Female athletes showed higher depression, more energy loss, and less motivation than did male athletes. ○ Male athletes’ libidos increased more than did the female athletes’. ○ Most athletes were unaware of online programs for mental and psychological support. PA: ○ Athletes practiced PA alone at moderate intensity for a period of 30–60 min daily. ○ Athletes showed some insecurities when they returned after lockdown. ○ Male athletes preferred sedentary activities such as watching television and playing video games.
Shanahan, 2020 [24]	To observe emotional distress in young adults related to COVID-19.	Perceived Stress Scale Social Behavior Questionnaire PROMIS^®^ Emotional Distress. Anger. Short Form	Mental Health: ○ Individuals reported higher stress and anger after the lockdown than before, although internalizing symptoms did not increase. Mental health related to PA: ○ Maintaining regular PA and positive reappraisal/reframing were associated with less distress. ○ Young adults whose well-being improved during the pandemic/lockdown tended to comment on the positive deceleration of their lives.
Shechter, 2020 [30]	To describe health care workers’ distress, coping behaviors, and preferences.	Electronic Qualtrics survey on mental health. 4-item PTSD ^p^ screen PC-PTSD ^q^: acute stress. PHQ-2 ^r^ GAD-2 Loneliness with a single item measure. Life Orientation Test-Revised: optimism with a single item. PSQI ^s^ and Insomnia Severity Index. COVID-19-related sources of distress. Coping behaviors. Psychological Screening.	Mental Health: ○ Participants worried about infecting their relatives. ○ Levels of distress were related to the health of relatives and friends, social distancing, lack of control over and/or insecurity related to coworkers’ COVID-19 situations, protective equipment, testing, and absence of national COVID-19 treatment guidelines. ○ Individuals screened positive for acute stress, depression, and anxiety. ○ sychological distress was higher for women, individuals living alone, and people with low incomes. PA: ○ The most used coping strategy was PA.
Stanton, 2020 [33]	To study the association between psychological distress and changes in health behaviors during the COVID-19 pandemic.	DASS 21 ^t^. AAS ^u^. AUDIT-C ^v^.	Mental Health: ○ Women showed higher levels of psychological distress. ○ Higher psychological distress was also found in those with the lowest incomes and those in the age group 18–45. PA: ○ Negative changes appeared. Mental health related to PA: ○ Higher levels of anxiety, depression, and stress were found in relation to PA changes, sleep, smoking, and alcohol habits.
Vasconcelos, 2020 [29]	To describe people’s behavior and how they were affected during COVID-19 quarantine.	Opinion survey.	PA: ○ Most participants were not exercising at all. ○ Most participants with poor housing conditions did not engage in PA. ○ Most participants with an income more than eight times the minimum wage were active. Mental health related to PA: ○ Those who exercised showed lower levels of stress and better sleep quality.
Wu, 2020 [27]	To study the changes in psychological factors and sleep status in COVID-19 front-line medical staff. To show evidence of exercise interventions to relieve psychological stress and improve sleep quality.	SCL-90 ^w^. SAS ^x^. SDS ^y^. PTSD Checklist- PCL-C ^z^. PSQI.	Mental Health: ○ Somatization, anxiety, depression, and terror scores were higher in the experimental group than in the control group. ○ The SAS scores were also higher in the experimental groups, whereas SDS and PCL-C scores were significantly higher in the control group.
Zachary, 2020 [6]	To quantify COVID-19 self-quarantine impacts on behaviors associated with weight gain.	Social network questions: body sizes of those whom the participants socialize. WALI ^aa^. PSS ^bb^ SIT-Q ^cc^. PAQ ^dd^.	Mental Health: ○ No correlation was found between PSS and weight gain. PA: ○ Respondents reported 2.7 ± 3.5 h/week of PA. ○ Sedentary behavior: 4.8 ± 3.3 h/day spent watching TV ^ee^ or playing video games, 2.5 ± 2.4 h/day on the computer. ○ Weight change was not predicted by total screen time. Relationship between PA and sedentary behavior time and weight: ○ There was a significant relationship between sleeping h/night and PA time on reported weight gain.
Zhang, 2020a [26]	To study the adverse impacts of the COVID-19 outbreak on mental health, understand its underlying mechanisms, and explore coping strategies.	IPAQ-S ^ff^. PSQI. DASS-21 BPAQgg	Mental Health: ○ Almost all participants were worried about the COVID-19 pandemic, reporting anxiety, stress, and depression. ○ No associations were found between COVID-19 death count, negative emotions, and global DASS scores. ○ With sleep quality as a mediator, the COVID-19 death count showed indirect impacts on general negative emotions, anxiety, and stress. PA: ○ Respondents reported 354.55 MET hh s/week of vigorous PA. ○ Men performed significantly more PA. Mental health related to PA: ○ Negative emotions improve with PA, especially at 2500 METs/week. ○ Depression was alleviated with PA. ○ Stress does not significantly improve with PA. ○ The dose-response curve indicates that an inadequate and/or excessive amount of PA worsened negative emotions.
Zhang, 2020b [28]	To assess health and well-being of adults living and working after one month of COVID-19 outbreak.	SF12 ^ii^ quality of life. Two composite scores of PCS ^jj^ and MCS ^kk^. K6 ^ll^. SWLS ^mm^.	Mental Health: ○ The distress and satisfaction with life scale scores were 1.41 and 3.22, respectively. ○ Quality of life scale: 16 participants scored 48.74 MCS. PA: ○ When people were active, they showed better well-being. ○ In the previous week, more than half of the sample had been active less than 1 h/day. ○ On the quality of life scale, 16 participants scored 49.55 in PCS. Mental health related to PA: ○ The severity of the COVID-19 outbreak in the participant’s location was significantly negatively associated with life satisfaction. For people who exercised 0.5 h/day or less, their life satisfaction was significantly positively associated with a more affected location.

^a^ MVC Mindfulness Virtual Community program, ^b^ CTB Cognitive Behavioral Therapy, ^c^ BPN Basic Psychological Needs, ^d^ COVID-19 Coronavirus disease 2019, ^e^ IPAQ International Physical Activity Questionnaire, ^f^ PA Physical Activity, ^g^ EMAs self-reported Ecological Momentary Assessments, ^h^ GAD-2 Generalized Anxiety Disorder-2, ^i^ GAD-7 Anxiety Disorder-7, ^j^ BREQ-3 Behavioral Regulations in Exercise Questionnaire, ^k^ NRS Nature Relatedness scale, ^l^ IPAQ-SF Short-form of International PA Questionnaire, ^m^ PGWBI Well Being Index, ^n^ PAR-Q Physical Activity Readiness Questionnaire, ^ñ^ REAP-S Rapid Eating and Activity Assessment for Participants short version, ^o^ DSM-5 Diagnostic and Statistical Manual of Mental Disorders-5, ^p^ PTSD Post-Traumatic Stress Disorder, ^q^ PC-PTSD Primary Care-Post-Traumatic Stress Disorder, ^r^ PHQ-2 Patient Health Questionnaire-2, ^s^ PSQI Pittsburgh Sleep Quality Index, ^t^ DASS 21 21-item Depression, Anxiety and Stress Scale, ^u^ AAS Active Australia Survey, ^v^ AUDIT-C Alcohol Use Disorder Identification. Test Consumption, ^w^ SCL-90 Symptom Checklist 90, ^x^ SAS Self-rating Anxiety Scale, ^y^ SDS Self-rating Depression Scale, ^z^ PCL-C Checklist-Civilian Version, ^aa^ WALI The Weight and Lifestyle Inventory, ^bb^ PSS Perceived Stress Scale, ^cc^ SIT-Q Sedentary behavior questionnaire, ^dd^ PAQ Physical Activity Questionnaire, ^ee^ TV Television, ^ff^ IPAQ-s International Physical Activity Questionnaire-Short, ^gg^ BPAQ Buss-Perry Aggressive Questionnaire, ^hh^ MET Metabolic Equivalent Task, ^ii^ SF12 Short Form-12, ^jj^ PCS Physical Composite Scale, ^kk^ MCS Mental Composite Scale, ^ll^ K6 Six-item Kessler psychological distress scale, ^mm^ SWLS Satisfaction with Life Scale.
